# SARS-CoV-2 antibodies recognize 23 distinct epitopic sites on the receptor binding domain

**DOI:** 10.21203/rs.3.rs-2800118/v1

**Published:** 2023-05-18

**Authors:** Jiansheng Jiang, Christopher T. Boughter, Javeed Ahmad, Kannan Natarajan, Lisa F. Boyd, Martin Meier-Schellersheim, David H. Margulies

**Affiliations:** 1 Molecular Biology Section, Laboratory of Immune System Biology, National Institute of Allergy and Infectious Diseases, NIH, Bethesda, MD 10892, USA; 2 Computational Biology Section, Laboratory of Immune System Biology, National Institute of Allergy and Infectious Diseases, NIH, Bethesda, MD 10892, USA

## Abstract

The COVID-19 pandemic and SARS-CoV-2 variants have dramatically illustrated the need for a better understanding of antigen (epitope)-antibody (paratope) interactions. To gain insight into the immunogenic characteristics of epitopic sites (ES), we systematically investigated the structures of 340 Abs and 83 nanobodies (Nbs) complexed with the Receptor Binding Domain (RBD) of the SARS-CoV-2 spike protein. We identified 23 distinct ES on the RBD surface and determined the frequencies of amino acid usage in the corresponding CDR paratopes. We describe a clustering method for analysis of ES similarities that reveals binding motifs of the paratopes and that provides insights for vaccine design and therapies for SARS-CoV-2, as well as a broader understanding of the structural basis of Ab-protein antigen (Ag) interactions.

## Introduction

Our ability to predict protein interactions is still very limited despite great progress in the application of computational methods for determining protein structures from amino acid sequence alone ^1 2^. This limitation is even more evident with regard to the interactions among highly variable immune receptor surfaces as dictated by Ab complementarity determining region (CDR) loops and the antigenic structures they bind. Accordingly, efforts directed toward providing systematic analyses or rational design strategies for Ab-Ag interactions need to incorporate experimentally determined structural data on specific Abs. Recent efforts in Ab design take advantage of segmental approaches ^3^ or extensive computational resources ^4,5^. Such hindrances emphasize the importance of incorporating as much information on naturally occurring specific Ab-Ag structures as possible. Here, we report a systematic structural analysis, taking advantage of the thousands of structures of SARS-CoV-2-derived proteins, including spike and various Ab complexes that have been determined to further our understanding of the fundamental mechanisms of the pathogenesis and neutralization of SARS-CoV-2 in the context of the human immune system. Many Abs have been reported to have potent neutralizing activity, preventing spike interaction with the cellular receptor, angiotensin converting enzyme (ACE) 2. Several Abs have been developed as therapeutics and have variable efficacy against variants of concern (VOC). Our analysis of available structures may aid in understanding which Abs may be of value for emerging variants and contribute to evolving strategies for prophylaxis, treatment, and immunization.

Ab-protein antigen (Ab-Ag) interfaces have been a focus of immunologists and protein chemists for more than 80 years ^6^, not only because of the important role of Abs in defense against infection ^7^, but also due to the general interest in understanding protein-protein interactions ^8^. High resolution structural analysis of protein-protein complexes, based initially on X-ray crystallography and more recently on cryogenic electron microscopy (cryo-EM), provides an objective basis for understanding not only the biophysical principles that determine affinity and specificity, but also for elucidating biological and evolutionary rules that govern immunological molecular recognition of foreign molecules and pathogens ^9,10^. With an ever-expanding database of detailed Ab-Ag structures, great attention has been directed to the characterization of such molecular interfaces, particularly as an understanding of the rules of engagement might permit rationalization of the reactivity of existing Abs, the design of Abs with new binding activities, and strategies for design of immunogens that might elicit more broadly neutralizing Abs ^11-13^.

The widespread infectivity, variance, and molecular characterization of the SARS-CoV-2 virus have provided a wealth of information concerning the functional and structural biology of the immune response. At the beginning of the SARS-CoV-2 pandemic, many laboratories accomplished detailed structural characterization of anti-RBD Abs and nanobodies (Nbs, single domain antibodies), leading to a classification of Abs based on the location of their footprints on the RBD surface. Initially, four classes of Ab were categorized, based on the orientation of the RBD bound and whether the Ab blocks infectivity or binding to the cellular receptor, ACE2 ^14^ (***Supplementary Table 1***). A receptor binding motif (RBM) has been defined as those RBD residues that specifically interact with ACE2 ^15^. Binding analysis of Nbs and human mAbs derived from patients along with a limited number of protein structures assigned five surface regions of the RBD reflecting its antigenic anatomy ^16^. Epitopic analysis was further extended by the definition of seven “communities” of Abs that bind to the RBD surface ^17^. Recent analysis of anti-RBD antibodies in the context of evolving escape mutations has taken advantage of these earlier classification schemes^18-21^.

Although these classification schemes have been valuable and adopted widely in the analysis of Abs as to how they bind to RBD and spike, particular Abs and Nbs may not be unambiguously classified (***Supplementary Figure 1***). The previous summaries were based on a relatively small number of available structures and focused on the relative superposition of the Abs in the complexes, rather than on a comparison of the epitopic contacts of the RBD surface. In particular, the original distinction between Class 1 and Class 2 seemed clear based on the initial structures. However, as more structural models became available, apparent inconsistencies arose. For example, Ahmad et al ^22^ determined that synthetic Nbs Sb16 and Sb45 contacted both Class 1 and Class 2 epitopic surfaces and approached the RBD from different angles. As more structures of Ab and Nb complexes are determined, it is apparent that an expansion of the initial classification scheme is warranted.

In this work, we focus on complexes of Abs and Nbs bound to the RBD of the spike protein to generate a comprehensive structural framework to further our understanding of Ab- and Nb-RBD recognition. Using a large database, we offer a structure-based classification exploiting quantitatively defined contacting amino acid residues on the RBD as well as a clustering analysis. These analyses reveal common characteristics of some 23 frequently contacted ES and the structural nature of the surfaces of the RBD that interact with Ab/Nb. We also systematically analyze the molecular features that define these antibodies and, by applying a rigorous evaluation of the surface features of the RBD that are seen by Abs and Nbs, generate general insights into the fundamental nature of Ab-Ag recognition. This analysis should facilitate the characterization of new anti-RBD antibodies as they arise.

## Results

### Identification of epitopic sites (ES)

To identify common features of ES of the RBD, we systematically investigated structures of Abs (as Fabs and Fvs, Ab fragments that confer antigen binding activity) and of Nbs (as VHH or synthetic library-derived sybodies) in complex with the spike protein or its RBD as collected in the CovAbDab ^23^ and the protein data bank (PDB)^24,25^. Abs and Nbs that bind the SARS-CoV-2 RBD are summarized in Table 1. As of 12/22/2022, a total of 6,746 Ab and 620 Nb sequences have been collected in the CovAbDab. Of the Abs, 6,321 are human, including those from vaccinees, and 390 derive from humanized mouse or phage display Ab libraries. For Nbs, 620 sequences derive from camelids (alpaca/camel/llama), of which 276 are from camelid-derived phage display libraries, some naïve, some immunized. Among these sequences, structural coordinates for only ~5% of the Abs and ~10% of Nbs were available in the PDB, and we compiled a non-redundant list of 340 Ab and of 83 Nb X-ray or cryo-EM structures (***Supplementary Table 2a & 2b***) which serve as the basis of our structural analysis.

**Fig. 1.**
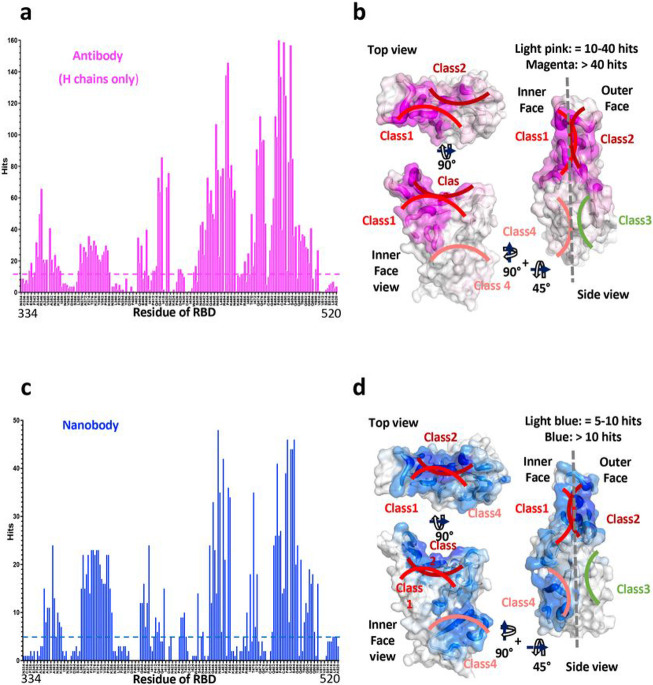
Number of contacts to RBD by Abs and Nbs. **a** Total number of contacts to each of the indicated RBD residues summed from all available X-ray and cryo-EM structures from Ab H chains. **b** Graphic depiction of number of contacts illustrated as footprint on the RBD and as putty heat map of RBD cartoon backbone. Top, inner face, and side views of RBD are shown. **c** Total number of contacts as in **a**, but for Nb contacts. **d** Surface footprint and putty heat map of Nb contacts as in **b**.

Evaluation of the biophysical properties that contribute to protein-protein interactions may be based on different criteria, including calculation of free energy terms of interacting residues ^26^, measurement of shape complementarity (Sc ^27^), and calculation of buried or accessible surface area ^28-32^. We elected to simplify this analysis first by calculating interatomic contacts between Ab (paratopic) and Ag (epitopic) residues at the interface because the biophysical basis of binding (due to charge, hydrophobicity, hydrogen bonding and van der Waals interactions) is reflected in such contacts. We calculated distances between Ab and Ag interface residues with a cut-off of 5.0 Å (see Methods) and we plotted the numbers of Ab (paratope) contacts as hits versus the residue number of the RBD (epitope) for the Ab heavy (H) (Figure 1a) and light (L) (***Supplementary Figure 2a***) chains individually, and also overall for both H and L chains together (***Supplementary Figure 2b***). We also plot the number of hits of the 83 Nbs to each RBD residue (Figure 1c). For 340 Abs, H chains contribute 5,623 contacts and L chains 3,107 (***Supplementary Table 3***). By comparison, for 83 Nbs, 1,836 contacts are observed. Thus, the number of contacts is ~25 per Ab and ~22 per Nb. Although the RBD residues bound by either Ab, H chain, or Nb are by and large, the same, the relative distribution of hits varies for several regions. In particular, the region from RBD residue 368 to 386 is recognized more frequently by Nbs, while other contiguous surfaces are seen equivalently (Figure 1a & 1c). The numbers of hits for Ab H chains are represented graphically as a heat map on the RBD surface in Figure 1b, and the heat maps for the Nbs are shown in Figure 1d.

**Fig. 2.**
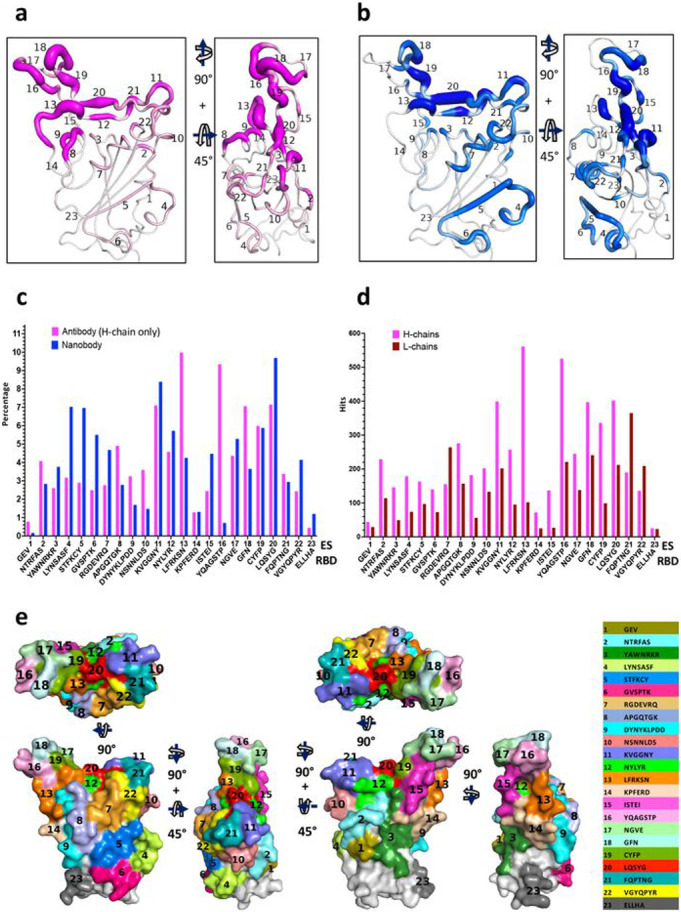
Distribution of Abs and Nbs on RBD surface. **a** Putty heat map of H chain of antibody with the definition of ES. The thickness of putty represents the number of hits. **b** Putty heat map of Nb with the definition of ES. **c** Distribution of Abs/Nbs on ES of RBD surface (percentage, %). Magenta represents Ab, blue represents Nb. **d** Comparison of antibody H chains and L chains on ES of RBD surface (by hit numbers). **e** ES surface area or footprint is illustrated by a color map of the RBD surface.

Several contiguous stretches of amino acids of the RBD that make Ab contact were apparent, although the frequency of hits varied considerably for different regions on the surface of the RBD. A fine-grained tabulation of regions of the RBD consisting of three to nine residues define each individual ES as shown in Table 2a. Each of these ES may be assigned to either of the four major classes identified earlier or to the RBM recognized by the ACE2 receptor (Table 2b). These regions include distinct secondary structural features such as strands, loops, turns, and helices (***Supplementary Movie 1a***), and represent contacts seen by few (<0.3 %) to many (>10%) Abs. Consideration of the secondary structural features (loops, turns, or short b strands) and the accessible surface area prompts the identification of 23 distinct contiguous sites, including regions encompassing residues 404 to 421 that had been overlooked in previous studies. The hit numbers are not evenly distributed over the RBD surface, and it is difficult to distinguish which binding sites belong to the previously defined Class 1 or Class 2 due to overlaps generated by the reduction of the three-dimensional surface to a two-dimensional plot. Figure 2a, b displays these ES on the RBD surface with the ES numbers for Abs (magenta) and Nbs (blue) respectively. The thickness of the putty cartoon indicates greater hit numbers. The computed accessible surface area (ASA) (see Methods) for each individual ES (Table 2a) ranged from ~100 Å^2^ to more than 500 Å^2^. The total buried surface area (BSA) is also computed for each of 340 Abs and 83 Nbs as in ***Supplementary Table 2a and 2b*** respectively. The values of BSA range from 106 Å^2^ (PDB 6XDG) to 1112 Å^2^ (PDB 7N64) for 340 Abs and from 444 Å^2^ (PDB 7JVB) to 1412 Å^2^ (7D2Z) for the 83 Nbs.

As an indication of the relative immunogenicity of each of the 23 ES, we tabulated the proportion of Abs and Nbs that recognized each site (Figure 2c). Approximately 7 to 11% of Ab H chains recognized ES11, 13, 16, 18, and 20, which represent ES contained within the previously defined Class 1 and Class 2 regions. In general, Nb recognition of specific ES was similar to that of Ab H chains, with the predominant recognition representing from 7 to about 10% of Nbs see Table 2a and Figure 2c, falling within Class 2 and Class 4. Notable differences in the predominant ES recognized by Abs and Nbs are that ES8, 13, 16, and 18 are more frequently seen by Abs while ES4, 5, 6, 7, 11, and 20 are more frequently identified by Nbs. For example, ES16 was recognized by 10% of Abs and by 0.16% of Nbs. This difference may be explained since ES16 forms a solvent exposed convex structure which may not be conducive to recognition by Nbs. By contrast, ES4, 5, and 6 form a contiguous patch, recognized more frequently by Nbs, a region that is not exposed to solvent in the complete spike when the RBD is in the down position. Thus, Nbs may be better able to access such hidden surfaces, perhaps because of their relatively small size (12kD compared to ~25 or 50 kD for Fv and Fab respectively or ~150 kD for complete bivalent IgG, with corresponding three-dimensional volumes) ^33^. Alternatively, since many Nbs were identified based on binding to isolated RBD, some epitopes identified from such screens may be partially hidden in the complete spike protein. In comparing L chains with H chains, as shown in Figure 2d, L chains generally contribute less to these ES. Nevertheless, L chains seem to preferentially contact ES7, 20 and 21. We note that some ES (e.g. ES7, 8, 9, and 23) could not be placed into the previous classification schemes and some sites overlap on Class 1 and Class 2 (i.e. ES12, 19, and 20). However, most of the 23 ES may be viewed within the four classes described by Barnes (Table 2b) ^14^. In addition, the RBM of the RBD ^15^ may be defined in terms of the ES that overlap the ACE2-RBD interface (i.e. ES8, 11, 12, 13, 16, 18, 19, 20, 21, and 22 (Table 2b)). With these 23 fine-grained ES, we extend the prior classification for Class 1 to now include ES8 and 9 (Table 2b). Each ES surface area or footprint is illustrated by a color map of the RBD surface (Figure 2d, ***Supplementary Movie 1b***). The sum of these 23 ES covers as much as 70% of the total accessible surface area (ASA) of the isolated RBD, illustrating the breadth of the human antibody response to RBD.

### Analysis of CDR loop contributions and epitope-paratope interactions

**Fig. 3.**
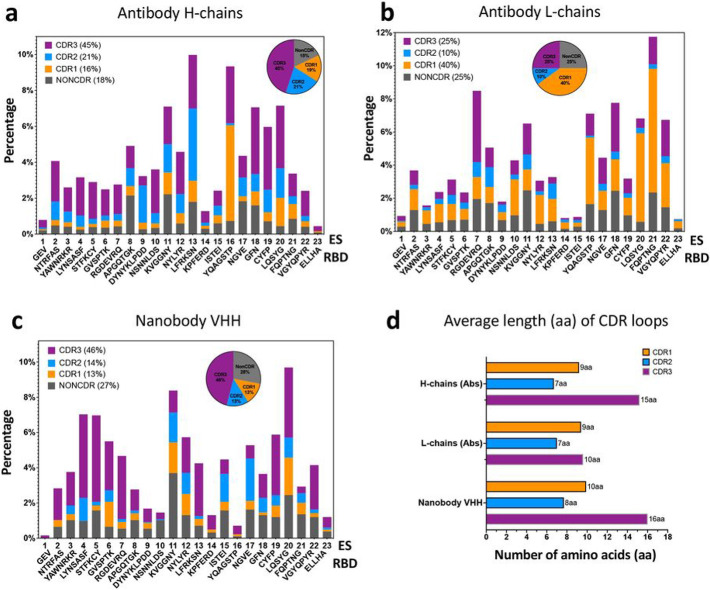
Distribution of CDR loops of contacts to RBD surface over ES. **a** Antibody H chains are plotted (percentage). Pie graph indicates the composition of CDR1 (16%, orange), CDR2 (21%, marine blue), CDR3 (45%, purple) and non-CDR (18%, gray) respectively. **b** Antibody L chains are plotted (percentage). Pie graph indicates the composition of CDR1 (40%, orange), CDR2 (10%, marine blue), CDR3 (25%, purple) and non-CDR (25%, gray) respectively. **c** Nanobody chains are plotted (percentage). Pie graph indicates the composition of CDR1 (13%, orange), CDR2 (14%, marine blue), CDR3 (46%, purple) and non-CDR (27%, gray) respectively. **d** Average length (in amino acids (aa)) of CDR loops extracted from the sequences (CovAbDab, ^23^, as of 12/20/2022) and used in this study. The averages are over 340 antibodies and 83 nanobodies respectively.

The CDRs in the hypervariable region of Abs play critical roles in recognizing antigens ^9,34,35^, and their variability in sequence and length facilitates interaction with distinct antigenic epitopes ^36^. We tabulated the number of contacts for each CDR loop or non-CDR residues of 340 H chains and L chains and 83 Nbs to each of the 23 ES. The contact percentages are summarized in Figure 3a, 3b and 3c respectively. The corresponding statistics are listed in ***Supplementary Table 3a, 3b*** and ***3c***. For Ab H chains (Figure 3a), CDR loops account for 82% of the contacts to ES (CDR1=16%, CDR2=21%, CDR3=45%), while only 18% of the contacts are from non-CDR residues. Interestingly, CDR1 of H chains play a major role in binding to ES16. For Ab L chains (Figure 3b), CDR1 loops play a major role (40%) in binding to RBD while CDR3 represent only 25% of the contacts. One explanation for the reduced the role of the CDR3 loop of L chains might be that their average length (10 aa for 340 Abs) is generally shorter than that of H chain CDR3 (15 aa for 340 Abs), see Figure 3d. For Nbs (Figure 3c), CDR represent 73% (CDR1=13%, CDR2=14%, CDR3=46%) of the contacts to the RBD surface, while 27% involve non-CDR residues. The average length of Nb CDR3 is 16 aa. Thus, for both Ab H chains and Nbs, CDR3 contributes the greater proportion of those residues that interact with the RBD, reflecting a major role for CDR3 in RBD recognition.

**Fig. 4.**
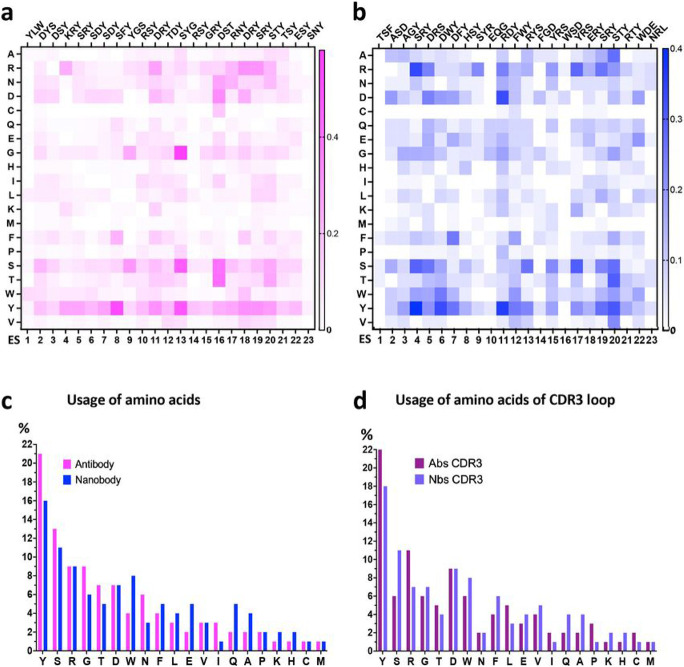
Distributions of amino acids of Abs/Nbs over ES. **a** Heat map of amino acids of Ab H chains on each ES, magenta indicates the frequency of the amino acids. **b** Heat map of amino acids of nanobody on each ES, blue indicates the frequency of the amino acids. Top triplets of amino acids are those most frequently observed amino of Abs/Nbs on each ES. **c** The usage of amino acids of antibody H chains (magenta) and nanobody (blue) in interacting with RBD is plotted in descending order (percentage). YSR are most frequently observed amino acids both for Ab and Nb. **d** Usage of amino acids in CDR3 loops (purple for Abs; light blue for Nbs). W of Nbs has relatively higher percentage in comparison to Abs both overall and for CDR3 loop.

We plotted the frequency of particular amino acids used by Abs and Nbs (paratopic residues) that interact with particular ES of the RBD for Ab H chains (Figure 4a) and for Nbs (Figure 4b). These are shown as heat maps. The residues listed on the top of the panel represent the most frequently contacting amino acids for the specific ES. The frequency of usage of each amino acid for Abs (pink) and Nbs (blue) is compared in Figure 4c. Tyrosine (Y), serine (S), and arginine (R) are the three amino acids most preferred for binding any ES of RBD (Figure 4c). Previous analyses of paratopic preferences for a wide range of Abs recognized a high frequency of tyrosine usage ^37^. We also observed that tryptophan is more frequently used in Nbs as compared with Abs (Figure 4c). The usage of CDR3 amino acids is plotted in Figure 4d. To illustrate the predominance of particular paratopic residues of the Ab H chains that contact specific ES, we also grouped these as WebLogo plots ^38^ (***Supplementary Figure 3***).

### Cluster analysis of epitopic sites and binding motifs

**Fig. 5.**
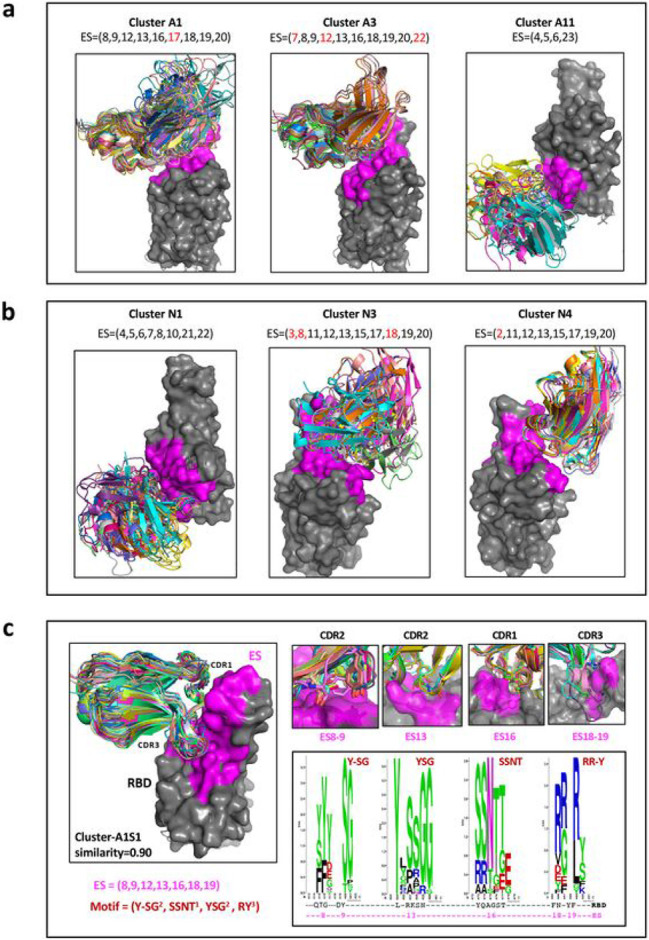
Identity of the similarity of the ES and clustering of Abs/Nbs (see Supplementary Table 4). **a** Illustration of three antibody clusters: A1, A3 and A11, each identifies a specific ES combination. Superimposed are members of the cluster on the RBD (only HV domains are shown for clarity). **b** Illustration of three nanobody clusters: N1, N3 and N4. RBD is are shown for clarity). **b** Illustration of three nanobody clusters: N1, N3 and N4. RBD is presented as gray surface, magenta indicates the binding areas (footprints) of ES of RBD. **c** A subset of the Cluster-A1, named A1S1, ES=(8,9,13,16,18,19) with similarity≥0.90, shows a strong binding motif on CDR loops. The members (28) of A1S1 are superposed on the RBD on the left panel. On the right top panel are shown the contacts between CDR loops and the binding sites (ES8-9, ES13, ES16, and ES18-19). On the right below panel, WebLogo plots show the amino acids from Abs binding to ES8-9, 13, and ES18-19 respectively. Y,S,G from CDR2 are favor binding to ES13 (RBD residues from 455-460); S,S,N,T of CDR1 favor binding to ES16 (RBD residues 455-459); and R and Y of CDR3 are favor binding to ES18-19 (RBD residues 485-491). **d** Clustering using AIMS ^43^. Here AX1 and AX2 are “principle components” of biophysical properties, or “mature information”.

Having identified the sets of ES bound by each Ab and Nb (see ***Supplementary Table 2a,2b***), we then grouped the Abs and Nbs by computation of the similarity of the ES recognized (see Methods). Similarity of a pair of ES sets is a value between 0 and 1 reflecting recognition of completely different (0) or identical (1) sites. This clustering method compares ES sets on the RBD without visualization of graphic models. Assigning a similarity threshold of 0.85 (see Methods) results in the identification of 33 distinct, non-overlapping, clusters for Abs, designated A1 to A33 (***Supplementary Table 4a***) and 10 distinct clusters for Nbs, N1 to N10 (***Supplementary Table 4b***). Although Abs within a single cluster bind the same subset of ES, they may, or may not address the RBD from the same angle or utilize CDR of the same length or composition. These differences are illustrated in Figure 5a for clusters A1, A3, and A11 for H chains and in Figure 5b for clusters N1, N3, and N4 for Nbs. The members of nanobody cluster N4 reveal a similar orientation because they have the same conformation and length of CDR loops. Abs or Nbs within the same cluster recognize the same contiguous RBD surface and are expected to compete sterically.

CDR loops contain sequence motifs for epitope recognition ^39-42^. To identify such motifs we analyzed a subset of interfaces from cluster A1, designated A1S1, that recognized ES with a similarity of ≥ 0.9. A1S1 consists of 28 members (cluster A1 has 56 members of similarity ≥ 0.85). All the members of A1S1 recognize the same ES set (ES8, 9, 12, 13, 16, 18, and 19) (Figure 5c), utilize the same CDR loops, and superpose well. Analysis of the residues of CDR1, 2, and 3 that contact the RBD indicated those residues that are preferentially utilized by this stringently selected cluster of Abs. For the binding motifs of CDR1, 2, and 3 of A1S1, the favored residues are summarized in a WebLogo plot (Figure 5c). Remarkably, Y, S, G, and T predominate for all CDR except CDR3 which exploits R in most instances. Thus, application of a more stringent ES similarity score helps to identify the preferred binding motif utilized by the Ab of the same subgroup. This stringent grouping of Abs and Nbs, based on high similarity score of their respective ES, may prove a useful adjunct in structure prediction based on amino acid sequence and antibody competition.

To extend the utility of our ES definitions, we set out to determine broad biophysical trends common among the Abs that cluster to each ES region. Using the automated immune molecule separator (AIMS) software ^43^, a tool which characterizes immune molecules without structural knowledge, we analyzed similar SARS-CoV-2-specific Abs. With this we identified 11 clusters which are designated as AIMS1, AIMS2, etc (Figure 5***d***). Not all Abs in a single AIMS cluster bind the same ES. However, AIMS6 and AIMS7 overlap as subsets of cluster A1 and have a similarity score of 0.85.

### Relation of ES and SARS-CoV-2 escape mutations

**Fig. 6.**
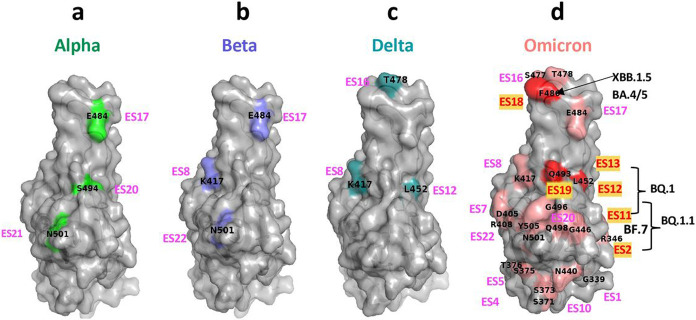
Ilustration of locations of variant mutations and associated ES on RBD surface. **a** Alpha variants. **b** Beta variants. **c** Delta variants. **d** Omicron variants and subvariants.

SARS-CoV-2 variants have evolved rapidly from Alpha, Beta, Delta, and Omicron with multiple mutations and deletions. The development of the latest Omicron subvariants can be traced from BA.1, BA.1.1, BA.2, BA.3, BA.4/5, and XBB.1 to XBB.1.5 and they incorporate as many as 30 mutations and deletions in their RBDs ^44-46^. ***Table 6a*** lists the mutations in these variants and the ES to which they map. Subvariants marked “X” have different substitutions at a given position. ***Table 6b*** lists the major Omicron subvariants and their associated ES. (For example, XBB.1.5 has substitutions of P and S for V445 and G446, respectively, which are contained in ES11, and substitution of S and Q for F490 and R493, respectively, which are in ES19). Similarly, XBB.4 preserves the same substitutions, but also substitutes R for L452 in ES12. Figure 6a illustrates the location of these variants on the RBD surface for Omicron and their mutation sites are matched to one or more of the 23 ES. Strikingly, Omicron escape mutations are distributed throughout several distinct ES of the RBD (***Table 6a***, Figure 6a-d), posing a formidable challenge in the design of new vaccines and therapeutic antibodies. Notably, mutations in ES3, 6, 9, 14, 15, and 23 have not yet been reported.

Our comprehensive analysis of RBD epitopes and their corresponding Ab paratopes offers the possibility of identifying currently approved SARS-CoV-2 therapeutic Abs that may be used to neutralize emerging SARS-CoV-2 variants and Omicron subvariants. The latest reported structures ^41,47-49^ describe some Abs that bind these subvariants. We can identify a number of Abs or Nbs that target particular ES sets that are either mutated or preserved in emerging variants. Those Abs/Nbs exhibiting multiple contacts to contiguous ES sites with concomitantly large buried surface area and high binding affinity deserve the greatest attention. Thus, using Ab/Nb structures already determined that target particular ES, we can model the effects of the variant mutations on antibody recognition.

Two examples illustrate this approach: the R346T RBD mutation in the subvariants BA.4, BA.5, BF.7 and XBB.1.5 lies within ES2 **(Table 2a, *Table 6a***, Figure 6a), and those Abs that recognize ES2 may be further evaluated for their ability to bind the mutants that harbor the R->T substitution. ***Supplementary Table 5a*** lists a number of Abs and Nbs whose structures are known that interact with ES2, and analysis of several Abs which may potentially resist the escape mutation (***Supplementary Figure 5a***). Specifically, the emergency use authorized (EUA) mAb S309 (one of three Fab modeled in PDB 7JX3) (sotrovimab)) may have neutralizing potency when combined with other antibodies to BA.1.1.529, BA.1, BA.2.75 subvariants ^50,51^. A second is the R486 mutation found in XBB.1 (R486S) and XBB1.5 (R486P) which is located in ES18 and 19 (F490 & R493). We identified a number of Abs and Nbs (***Supplementary Table 5b***) that have multiple contacts with ES17, 18, and 19, such as for COVOX-45, which preserves those to P486 from the main-chain of the CDR3 loop. Also, the nanobody Nb-2-67 makes multiple hydrogen bonds to maintain contact with ES18 (***Supplementary Figure 5b***).

Our analysis of ES recognized by Abs and Nbs and the identification of specific ES affected by mutations in VOC provides an explanation for the ineffectiveness of some Ab that have been tested therapeutically. One example, Evushield^™^, which consists of two Abs, tixagevimab (AZD 8895) and cligavimab (AZD 1061) illustrates this point. These Ab have been studied by X-ray crystallography (tixagevimab, PDB 7L7D, and cligavimab 7L7E ^52^) and by cryo-EM ^53^. By our analysis, tixagevimab interacts with ES13, 16, 18, 19, and 20 and cligavimab with ES2, 10, 11, and 12. As shown in ***Table 6a***, residues in every one of these ES are mutated in the Omicron variant. This then explains the lack of beneficial effect of Evushield^™^ and supports a molecular basis for the recent revision of its EUA by the FDA (https://www.fda.gov/drugs/drug-safety-and-availability/fda-announces-evusheld-not-currently-authorized-emergency-use-us). This highlights the importance of our analysis of ES bound by Abs and Nbs.

## Discussion

The enormous world-wide effort to elucidate the mechanistic underpinnings of the immune response to SARS-CoV-2 has provided deep insight into aspects of the B cell and T cell responses to infection and immunization and has contributed to ongoing strategies for therapy and prevention. Here, we have taken advantage of the ever-increasing structural database of anti-SARS-CoV-2 Abs and Nbs to analyze the three-dimensional features that are described by X-ray and cryo-EM structures of Ab and Nb complexes with the RBD of the virus, either alone or in the context of the full spike protein. We have developed several analytical computational tools described in detail in the methods that allow the tabulation and analysis of molecular contacts and ES between the Abs/Nbs and the RBD. These provide a convenient avenue for querying and comparing the binding sites and interactions of particular Abs/Nbs and will support additional queries as the Cov-AbDab and PDB entries increase. This has permitted the categorization of the epitope-paratope interactions and molecular surface characteristics that lend themselves to recognition by Abs and the recurrent structural motifs of the CDR residues of the Abs/Nbs. This identification of 23 ES derives from evaluation of a large number of Ab/Nb-RBD and Ab/Nb-spike structures and their interface contacts, and thus surpasses analyses based on amino acid sequence or gross structural comparison alone. Our method of clustering ES sites with various stringencies, and independently of the antibodies that recognize them, offers an additional tool towards the goal of prediction of CDR sequences that recognize particular epitopic sites.

Of some 340 Abs and 83 Nbs, our analysis indicates that the 23 ES on the RBD characterized in part by secondary structural features may be recognized at different frequencies. This fine-grained analysis of the RBD surface reveals that as many as 10% of Abs may recognize common features such as those of ES16 as seen by Abs, or of ES11 as seen by Nbs.

Understanding the biophysical or structural characteristics of antigenic or immunogenic sites on protein antigens has been a subject of considerable interest for many years, beginning with efforts to understand common sites seen by heterogeneous Abs and further refined as monoclonal Abs have been studied ^6,34,36,37,54^. Recent efforts have identified common motifs that human antibodies exploit to bind similar epitopes ^55^. Consistent features of antigenic sites include hydrophobicity, accessibility, and segmental mobility as well as sequence dissimilarity to the Ab-producing organism (tolerance). Here we have taken the opportunity to investigate a large number of Abs and Nbs for which the antigenic site of a single protein is defined at high resolution by structural criteria. Although non-random factors may contribute to biases in the available database. several important consistent conclusions may be drawn: 1) common sites are recognized by a proportion of Abs or Nbs approaching 10%; 2) several major surfaces of the RBD have not been addressed by either Abs or Nbs; and 3) some sites are favored by either Abs (e.g, ES16 and ES18) or by Nbs (e.g., ES4 and ES5). This latter phenomenon may reflect germline VH gene preferences in the human (as suggested ^56^) or the well-recognized characteristic of Nbs, whose relatively long CDR3 loops are capable of exploring concave surfaces ^57^.

Our analysis suggests that several regions of the RBD may be particularly important to incorporate into peptide-based immunogens (such as ES11, 13, 16, and 18) and that further generation vaccines might pay particular attention to new viral variants that affect these sites. Alternatively, Ab therapies may benefit from a focus on those reagents that recognize both common antigenic sites as well as those that are rarely identified. Although our analysis here has been confined to Abs/Nbs that recognize the RBD of the spike protein of SARS-CoV-2, this approach may, in principle, be applied to a variety Abs/Nbs directed against proteins of pathogenic organisms.

## Methods

### Databases

Covid antibodies and nanobodies were culled from the Coronavirus Antibody Database, Cov-AbDab (http://opig.stats.ox.ac.uk/webapps/covabdab/)^23^ and coordinates of three-dimensional models were taken from the protein database (PDB)( https://www.rcsb.org/, https://rcsb.org/covid19/)^24,25^.

### Software

All analyses were performed with our **EPI (E**pitope**-P**aratope **I**nteraction) software package of mixed scripts of C-shell, perl and python. **EPI** software is available at https://github.com/jiangj-niaid/EPI/. Contact distances were calculated based on scripts from **CNS 1.3** (http://cns-online.org/v1.3/) ^58^, using a cut-off of 5.0 Å. Buried surface area (BSA) ^31,59,60^ was calculated with **PISA** (**P**roteins, **I**nterfaces, **S**tructures and **A**ssemblies ^31^), and accessible surface area (ASA) ^32,61-64^ with **CNS.**

The clustering method used in EPI is based on the ES (i.e. RBD binding sites) not amino acid sequences of Abs or Nb2. The numbers of ES (1-23) are converted to a corresponding string of 23 letters from “a” to “w” and the similarity between sets of ES is computed using the Normalized Edit Distance that was developed from Hamming Distance^65^ and Levenshtein Distance ^66^. A similarity of 1 indicates that the two strings or two ES sets are identical; a similarity of 0 indicates that the two strings or ES sets are completely different. The similarity is then calculated for pairwise combinations of all Abs or Nbs based on their ES sets. Abs or Nbs can be clustered by imposing a similarity threshold. For 340 Abs we tested similarity thresholds from 0.50 to 0.99 at 0.05 intervals and found that a similarity threshold of 0.85 yielded 33 clusters that covered all Ab without overlap between clusters. For 83 Nbs a similarity threshold of 0.85 yielded 10 clusters. We also provide a program with which users can make inquire for a particular ES combination, PDB ID, antibody name, or Class 1-4 designation, at a given similarity threshold.

The AIMS analysis package used for biophysical clustering of antibody sequences can be found at https://github.com/ctboughter/AIMS, including generalized Jupyter Notebooks and a Python-based GUI for the replication of the results presented herein or for the application of this analysis to novel datasets. Detailed descriptions of the foundational concepts critical for this analysis and the instructions for use can be found at https://aims-doc.readthedocs.io.

Figures for structural models are generated by using PyMOL ^67^ (https://pymol.org/2/). Sequence logo figures were generated with WebLogo (https://weblogo.berkeley.edu/) ^38^. Sequence alignments were made with Clustal Omega (https://www.ebi.ac.uk/Tools/msa/clustalo/) ^68^. Graphic plots were generated with Prism 9 (https://GraphPad.com).

## Figures and Tables

**Table 1.** Summary of sequences and structures of anti-SARS-CoV-2 antibodies and nanobodies. The sequences and origin/source are collected in CovAbDab ^23^, as of 12/20/2022. The number of structures of antibodies and nanobodies in complex with RBD or spike protein are downloaded from PDB.

Origin/Source	Sequences	Structures * (PDB ID)
**Total Antibodies**	**6746**	**340**
Human (patient or vaccinee)	6321	250
Mouse (immunized/humanized)	165	40
Phage Display Library or engineered	225	33
Undefined	35	25
**Total Nanobodies**	**620**	**83**
Immunized (Alpaca/Camel/llama^#^)	332 (123/18/189)	50 (16/5/27)
Phage display library	276	23
Undefined	14	12

* Unique non-redundant structures determined either by X-ray or cryo-EM as listed in the PDB.

# This includes two sequences/structures from mice engineered to express llama Nb genes ^64^.

**Table 2.** Definitions of Epitopic Sites (ES) seen by Abs and Nbs. **a** RBD residue range for each ES is indicated, along with the amino acid sequence, secondary structural features (as determined by DSSP ^65^), accessible surface area (ASA) (see Methods) of the contacting residues, and percentage of Ab H chains and Nbs. **b** correlation of ES with Class definitions by Barnes ^14^ and with receptor binding motif (RBM) ^15^.

a	ES	RBD Residue (Range)	Amino Acid Sequence	Structural feature	ASA (Å^2^)	Ab (H chain) (%)	Nbs (%)
	1	339-341	GEV	α-helix	155	0.78	0.16
	2	343-349	NATRFAS	loop	455	4.07	2.83
	3	351-357	YAWNRKR	3_10_ ->β-strand	410	2.60	3.76
	4	368-374	LYNSASF	α-helix->loop	469	3.17	7.03
	5	375-380	STFKCY	β-strand	287	2.90	6.97
	6	381-386	GVSPTK	Loop->3_10_	381	2.49	5.50
	7	403-409	RGDEVRQ	3_10_	321	2.76	4.68
	8	411-417	APGQTGK	loop	342	4.91	2.78
	9	420-428	DYNYKLPDD	α-helix->loop	371	3.24	1.69
	10	437-443	NSNNLDS	β-strand->α-helix	378	3.59	1.47
	11	444-449	KVGGNY	loop/strand	457	7.10	**8.39**
	12	450-454	NYLYR	β-strand	194	4.57	5.72
	13	455-460	LFRKSN	loop	448	**9.9**8	4.25
	14	462-467	KPFERD	loop	470	1.28	1.31
	15	468-472	ISTEI	loop	384	2.44	4.47
	16	473-479	YQAGSTP	β-strand->loop	466	**9.34**	0.71
	17	481-484	NGVE	loop	404	4.36	5.28
	18	485-487	GFN	loop	275	7.06	3.65
	19	488-491	CYFP	β-strand->loop	181	5.98	5.88
	20	492-496	LQSYG	β-strand	126	7.15	**9.69**
	21	497-502	FQPTNG	loop	307	3.38	2.94
	22	503-509	VGYQPYR	3_10_->β-strand	253	2.42	4.14
	23	516-520	ELLHA	loop	548	0.44	1.20
b							
	Class	Epitopic Sites (ES)					
	1	7, 8, 9, 13, 16, 18, 21, 22					
	2	10, 11, 12, 15, 17, 19, 20					
	3	1, 2, 3, 14 , 23					
	4	4, 5, 6					
	RBM	8, 11, 12, 13, 16, 18, 19, 20, 21, 22					

**Table 3.** Relation of ES to SARS2-CoV-2 escape mutations. **a** Major mutations with the main lineage of variants of SERS-Cov-2 and corresponding ES site. “X” column indicates the amino acids substitution of the sub-variants of Omicron. **b** Latest mutations in the major lineage of sub-variants of Omicron and corresponding ES site.

a	Variant	Alpha	Beta	Delta	Omicron	Sub (X)	ES
	**Mutations**				G339D		**1**
					R346X	T,K,E,I,S	**2**
					S371L		**4**
					S375F		**4**
					T376A		**5**
					D405N		**7**
			K417N	K417N	K417N		**8**
					N440K		**10**
					K444X	T,R,N,M	**11**
					V445X	A,P	**11**
					G446X	D,S	**11**
				L452R	L452X	Q,R,M	**12**
					N460X	K,S,Y	**13**
					S477N		**16**
				T478K	T478K		**16**
		E484K	E484K		E484A		**17**
					F486X	I,V,P,S	**18**
					F490X	I,L,S,V	**19**
					R493X	Q,L	**19**
		S494P					**20**
					G496S		**20**
					Q498R		**21**
		N501Y	N501Y		N501Y		**21**
					Y505H		**22**

b												
	ES	ES2	ES11			ES12	ES13	ES18	ES19		Structures	
	**Mutations**	**R346X**	**K444X**	**V445X**	**G446X**	**L452X**	**N460X**	**F486X**	**F490X**	R493*	**unligand**	**Complex**
	**B.1.1.529**				S					Q	7t9j	
	**BA.2.13**					M				R	7xnr	
	**BA.2.75**				S		K			Q	7yqv	
	**BA.3**				S					R	7xiy,7xiz	7wtf;7wtg;7wth
	**BA.4.6**	T				R		V		Q	7xnq,7xns	
	**BA.5.1.26**	T				R		V		Q		
	**BA.5.2**					R		V		Q		
	**BF.7**	T				R		V		Q		
	**BQ.1**		T			R	K	V		Q		
	**BQ.1.1**	T	T			R	K	V		Q		
	**XBB.1**	T		P	S		K	S	S	Q		
	**XBB.1.5**	T		P	S		K	P	S	Q		
	**XBB.4**	T		P	S	R	K	S	S	Q		

## Data Availability

All data generated for analysis in this study has been published on GitHub at https://github.com/jiangj-niaid/RBD-SARS2/.
